# The influence of variability in species trait data on community‐level ecological prediction and inference

**DOI:** 10.1002/ece3.2385

**Published:** 2016-08-14

**Authors:** Karen M. Alofs

**Affiliations:** ^1^ Department of Ecology and Evolutionary BiologyUniversity of TorontoTorontoONCanada

**Keywords:** Body size, fish, phylogenetic signal, predator introduction, range shifts

## Abstract

Species trait data have been used to predict and infer ecological processes and the responses of biological communities to environmental changes. It has also been suggested that, in lieu of trait, data niche differences can be inferred from phylogenetic distance. It remains unclear how variation in trait data may influence the strength and character of ecological inference. Using species‐level trait data in community ecology assumes intraspecific variation is small in comparison with interspecific variation. Intraspecific variation across species ranges or within populations may lead to variability in trait data derived from different scales (i.e., local or regional) and methods (i.e., mean or maximum values). Variation in trait data across species can affect community‐level relationships. I examined variability in body size, a key trait often measured across taxa. I collected 12 metrics of fish species length (including common and maximum values) for 40 species from literature, online databases, museum collections, and field data. I then tested whether different metrics of fish length could consistently predict observed species range boundary shifts and the impacts of an introduced predator on inland lake fish communities across Ontario, Canada. I also investigated whether phylogenetic signal, an indicator of niche‐conservativism, changed among measures. I found strong correlations between length metrics and limited variation across metrics. Accordingly, length was a consistently significant predictor of the response of fish communities to environmental change. Additionally, I found significant evidence of phylogenetic signal in fish length across metrics. Limited variation in length across metrics (within species), in comparison with variation within metrics (across species), made fish species length a reliable predictor at a community‐level. When considering species‐level trait data from different sources, researchers should examine the potential influence of intraspecific trait variation on data derived by different metrics and at different scales.

## Introduction

Ten years ago, McGill et al. (2006) suggested “rebuilding” community ecology by examining how species traits control the relationship between a species' niche and its environment. Since then, species traits have been used to build mechanistic inferences on topics ranging from community assembly (HilleRisLambers et al. 2012), to biodiversity patterns (Meynard et al. 2011; Swenson et al. 2011), to ecosystem functioning (de Bello et al. 2010; Flynn et al. 2011), to the impacts of environmental change (Angert et al. 2011). Further, despite criticism, phylogenetic distance has been used as a proxy for differences in species traits or niche differences (HilleRisLambers et al. 2012; Gerhold et al. 2015). This use assumes phylogenetic signal or phylogenetic niche‐conservativism (although meta‐analyses have suggested phylogenetic signal is not as common as previously believed; Kelly et al. 2014).

Intraspecific trait variation is known to have significant ecological and evolutionary effects (Bolnick et al. 2011) and not incorporating such variation into trait‐based studies can influence the accuracy of predictions in community ecology (Albert et al. 2012; Violle et al. 2012). However, data quantifying community‐wide intraspecific trait variation are often not available or difficult to attain, particularly for studies at regional scales. Therefore, species‐level trait values are often substituted. The use of species‐level trait data for community‐level inference relies upon the assumption that intraspecific variation is low, particularly in comparison to interspecific variation (McGill et al. 2006). We know, however, this is not always the case, particularly for species distributed across environmental gradients (Albert et al. 2010). In part due to intraspecific trait variation, species trait data may vary significantly when gathered from field observations, experiments, literature (e.g., field guides or atlases) or from taxonomically or regionally distinct online databases. Few studies have examined the consistency of species trait data across sources (Fitzsimmons 2013; Kazakou et al. 2014); although some work has been done on sensitivity to missing data and variable sampling effort (Pakeman 2014; Sandel et al. 2015). Trait values are often presented as “mean,” “common,” or “maximum” values and sensitivity to type of metric for single traits remains unclear. Further, individual sources offer little detail on variation in species traits through space or time, although the importance of this variation has been recognized (Fitzsimmons 2013; Kazakou et al. 2014).

I use species data for a single trait, body size, collected from six sources (online, print, collection and field based) to examine how variability in species‐level trait data influences trait‐based predictions in community ecology. I also test whether variability in trait data influences our ability to detect phylogenetic signal and infer the usefulness of phylogenetic relatedness as a proxy for ecological relatedness. Size (e.g., length, volume or mass) is perhaps the most commonly measured individual trait across taxa. It influences many aspects of ecology and evolution and is correlated with other species traits including those which determine metabolism, reproductive rate, dispersal, and trophic interactions (LaBarbera 1989; Brown et al. 2004; Woodward et al. 2005). Further, body size has been used to infer the direct and indirect impacts of human activities including hunting and fishing (Jennings and Blanchard 2004; Fenberg and Roy 2008), land‐use changes (Mulder and Elser 2009), introduced species (Ness et al. 2004; Alofs and Jackson 2015), and climate warming (Gardner et al. 2011; Alofs et al. 2014).

Here, I focus on how variation in freshwater fish body size, particularly species length metrics, may influence community‐level inference. Many freshwater fishes demonstrate significant intraspecific trait variation within populations, across species ranges and along environmental gradients (e.g., Einum and Fleming 2002; Heins et al. 2004; Blanck and Lamouroux 2006; Gutowsky and Fox 2012). Across species, length is related to a range of life‐history traits including fecundity and longevity (Winemiller and Rose 1992; Alofs et al. 2014). Alofs et al. (2014) demonstrated there was a significant relationship between average body length in Ontario, Canada (as reported by Holm et al. 2009), and the magnitude of northern range boundary shifts by warm‐ and coolwater‐adapted fishes in that province over about 30 years. Specifically, larger predatory fishes demonstrated greater poleward shifts than smaller prey species (including both warm‐ and coolwater‐adapted fishes). In Ontario, resident populations of small prey fishes also appear to be more vulnerable to the introductions of these large range‐expanding predators as they establish in northern lakes (Alofs and Jackson 2015). Particularly, average Ontario length is significantly related to the relative risk imposed by introductions of *Amboplites rupestris* (Rock Bass), a gape‐limited predator.

Intraspecific variation across species ranges and within populations can produce variability in trait data measured at different scales or by different methods. The relative amount of intraspecific variation, in contrast to interspecific variation, may control the usefulness and reliability of trait data. I used data on inland lake fish communities across Ontario (from Alofs et al. 2014 and Alofs and Jackson 2015) and a phylogeny of Ontario fish communities (Doyle 2013) to test the influence of variability in species length metrics on community‐level inference. I hypothesized that, given variability across length metrics, the strength of ecological prediction and inference would vary by the method and the scale of data collection. Specifically, prediction and inference would be strengthened by using (1) species averages or common estimates of traits rather than species maximums or records, which may represent outliers, and (2) using species data derived in Ontario rather than at larger scales. I test this idea using 12 metrics of fish length (collected from a variety of sources) to examine (1) the relationship between size and the extent of northern range boundary shifts by fishes, (2) the relationship between size and the vulnerability of common species to the introduction of a predatory fish, and (3) evidence of phylogenetic signal in size.

## Materials and Methods

### Data

**Figure 1:**
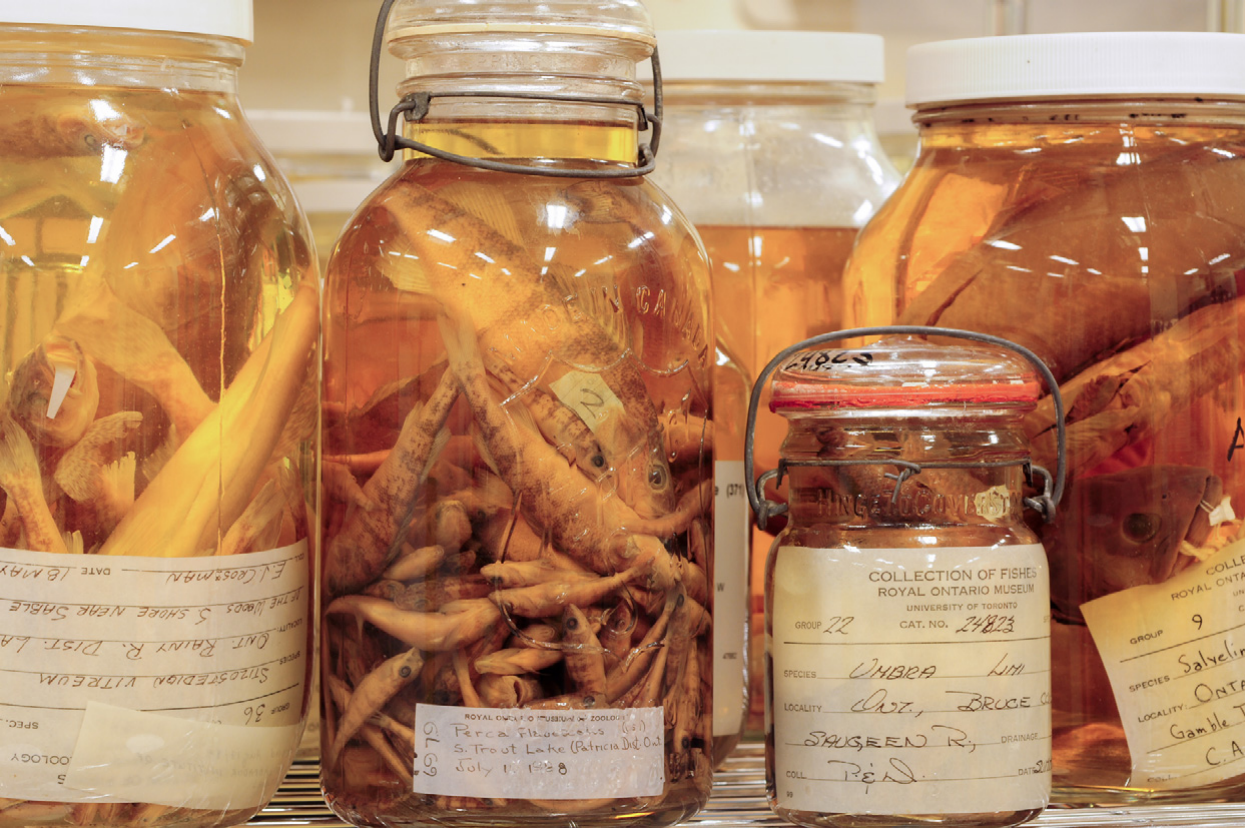
Fishes in the collection of the Royal Ontario Museum. The maximum total length (mm) from catalogued Ontario specimens was one of the metrics used in this study.

I collected 12 different metrics of fish length for 40 species (Table 1, Supporting Information, Appendix S1; species were selected based on community‐level analysis described below) from two online sources, two print sources, the Royal Ontario Museum collection (Fig. 1) and field data collected by the Ontario Ministry of Natural Resources and Forestry during their Broad‐scale Monitoring (BsM) program (Sandstrom et al. 2010). The BsM field data include length data for more than 250 thousand individual fishes from standardized sampling of 745 lakes across Ontario. The 12 length metrics included five “average,” “common,” or “median” and seven “maximum” or “record” metrics. Metrics referred to as “average” or “common” in print and online databases were not clearly described in sources and are likely to be based on expert opinion rather than calculated from sampled data (E. Holm, Royal Ontario Museum, pers. comm.). Most metrics were based on measures of total length (from tip of snout to tip of closed caudal fin); however, during BsM, fork length (from tip of snout to center of open caudal fin) was measured. Fork length is correlated with total length (Carlander and Smith 1945) although shorter than total length particularly for species with lobed caudal fins, and at most equal to total length.

**Table 1:** Description of 12 measures of fish length collected from various sources

Code	Source	Database type	Measure type	Length measure
FB.MAX	FishBase	Online	Maximum	Total length^a^
FB.COM			Common	Total length
FT.MAX	FishTraits Database	Online	Maximum	Total length
FC.MAX	Freshwater Fishes of Canada (Scott and Crossman 1998)	Print	Maximum	Total length
FC.COM			Common (or maximum of common range)	Total length
FO.WORLD.MAX	Freshwater Fishes of Ontario (Holm et al. 2009)	Print	World record	Total length
FO.ON.MAX			Ontario record	Total length
FO.ON.AVG			Ontario average	Total length
ROM.MAX	Royal Ontario Museum	Collection	Maximum in all catalogued lots	Total length
BSM.MAX	OMNRF Broad‐scale Monitoring	Field	Absolute maximum of all measured fish	Fork length
BSM.AVG			Maximum per lake averaged across lakes	Fork Length
BSM.MED			Median of all measured fish	Fork Length

a Fork Length was reported for Walleye.

Five missing values were substituted from alternative sources to complete the data set: world record length for *Coregonus artedi* was not reported in the *Freshwater Fishes of Ontario*, and thus, the Ontario record, maximum in the data set, was used. The common length of *Osmerus mordax* was not reported in *Fishbase* and the average common length from the *Freshwater Fishes of Canada* was substituted. The *Freshwater Fishes of Canada* did not report a maximum length for *Chrosomus neogaeus, Notropis heterodon* and *N. volucellus* and the world record reported in the *Freshwater Fishes of Ontario* was substituted.

I collected the relative northern range boundary shift of 13 species calculated as the change in mean latitude of most northern 20% of occurrences in a data set of 1527 lakes across Ontario that were sampled during a historical and contemporary period ~30 years apart (from Alofs et al. 2014; Table 1, Supporting Information, Appendix S1). I also collected data on the vulnerability of 29 species to Rock Bass introductions; the relative risk ratio associated with these predator introductions was calculated from two‐by‐two contingency tables created from a data set of 1551 Ontario lakes also sampled in two time periods (from Alofs and Jackson 2015; Table 1, Supporting Information, Appendix S1). The relative risk ratio measures the impact of introduction as the probability of each resident species “loss” (presence during a historical survey and absence during a contemporary survey) given introduction and establishment of *Amboplites rupestris* (Rock Bass) over the probability of loss given no establishment, that is, background variation. Finally, I used the phylogenetic tree of 26 species occurring in lakes on Manitoulin Island, Ontario, published by Doyle (2013); species indicated in Table 1, Supporting Information, Appendix S1).

### Analysis

I calculated correlations between each of the 12 metrics of fish length. To compare variation among metrics of length to interspecific variation in length, I calculated the coefficient of variation (CV) for each species (across metrics) and each metric (across species). For 13 species, I fitted separate linear regression models of relative northern range boundary shifts against each of the 12 length metrics. For 29 species, I fitted similar linear regression models of the relative risk associated with predator introductions against each of the length metrics. For all of these models, I examined standardized (z‐score) effect sizes, *R* ^2^, and *P*‐values. All size metrics and the relative risk ratios were natural‐log‐transformed for analysis. All analyses were performed with R 3.1.2 (R Development Core Team 2014).

To test for evidence of phylogenetic signal across 26 species, I calculated four commonly used indices (described and compared in Münkemüller et al. 2012): Moran's *I*, Abouheif's *C*_mean_, Pagel's *λ* and Blomberg's *K*. Moran's *I* and Abouheif's *C*_mean_ are autocorrelation indices and not based on an evolutionary model. Stronger phylogenetic signal is indicated by greater deviation from zero by these estimates. Pagel's *λ* and Blomberg's *K* are based on a Brownian motion model of trait evolution. Similar to *I* and *C*_mean_, *λ* and *K* approach zero with phylogenetic independence. The upper limit of *λ* is near one, while *K* may exceed one indicating greater phylogenetic signal than expected with Brownian motion. Moran's *I* and Abouheif's *C*_mean_ were estimated and their significance in comparison with random trait variation tested with Monte Carlo simulations (999 randomizations) using the aboufeif.moran function in the adephylo package. Pagel's *λ* and Blomberg's *K* were estimated and their significance was tested (999 randomizations) using the phylosig function in the phytools package. The significance of *K* was tested using a randomization test, while that of *λ* was tested by a likelihood ratio test.

## Results

**Figure 2:**
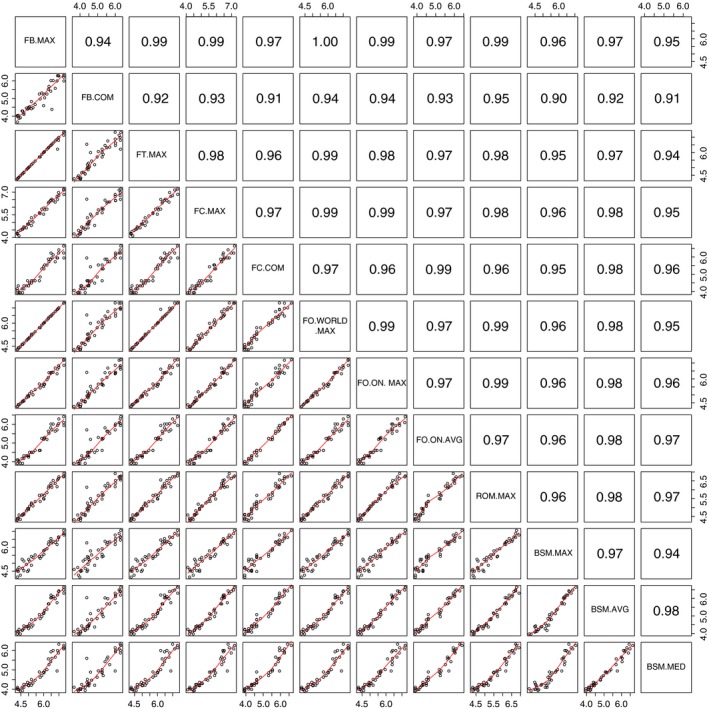
Correlation matrix for 12 metrics of fish species length for 40 species.

Length data were highly correlated between the 12 metrics and across the 40 fish species *I* considered (Fig. 2). Correlations ranged from *r* = 0.9089 between the common length reported on *FishBase* and median length recorded in BsM field data to *r* = 0.9998 between the maximum length reported on *FishBase* and the world record reported in the *Freshwater Fishes of Ontario*. Variation among metrics was greater for larger species (higher CV across metrics; Supporting Information Appendix S1). The mean CV of length among metrics within species (range 0.15–0.52, mean = 0.33, SD = 0.09) was less than half the variation in length among species within metrics (range 0.80–0.93, mean = 0.86, SD = 0.05).

**Figure 3:**
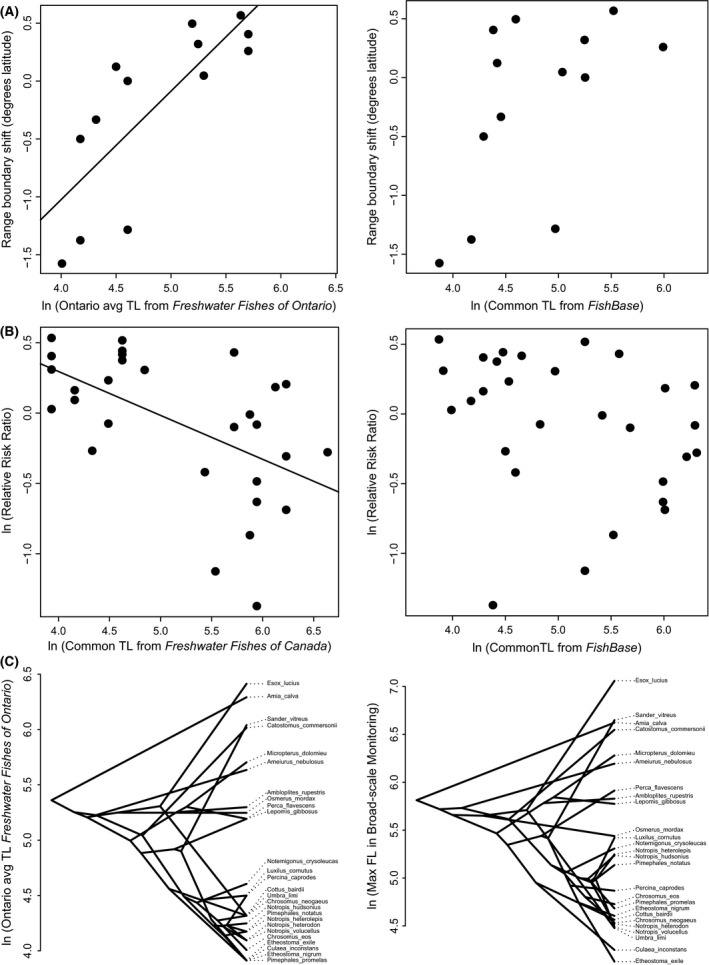
The strongest (left) and weakest (right) relationships between fish species length and (A) the magnitude of northern range boundary shifts or (B) the relative risk on resident populations imposed by predatory Rock Bass introductions. Followed by (C) traitgrams for the measures with relatively strong (left) and weak (right) phylogenetic signal. Traitgrams plot species by their relative size along the *y*‐axis and connect species with an underlying phylogenetic tree. More crossed lines indicate a trait is more randomly distributed indicating less phylogenetic signal.

All metrics but the common length reported on *FishBase* were significant predictors of both range shifts and the impact of predator introduction (at the *α *= 0.05 level; Table 2, Fig. 3). Length was positively related to range shifts with standardized effect sizes (*β*s) ranging from 0.55 to 0.98. Length had the strongest effect on predicted range shifts when calculated with field BsM data. Length was negatively related to the impact of predators; standardized effect sizes varied between −0.22 and −0.27, with the exception of common length reported on *FishBase* (*β *= −0.15, but nonsignificant). Aside from this metric, there was little change in the amount of variation in either response explained by length metrics (*R* ^2^ values in Table 1). Average Ontario length reported in the *Freshwater Fishes of Ontario* was the strongest predictor of (explained the most variation in) northern range boundary shifts. Average maximum length reported in the *Freshwater Fishes of Canada* was the strongest predictor of the impact of *A. rupestris* introductions.

**Table 2:** The *R* ^2^ and *P*‐values from regressions of 12 measures of fish species length as predictors of range boundary shifts and the impact of predator introductions

	Range boundary shift (df = 11)			Impact of predator introduction (df = 27)		
	*β*	*R* ^2^	*P*	*β*	*R* ^2^	*P*
FO.ON.AVG	0.76	0.62	0.001	−0.25	0.26	0.005
BSM.AVG	0.78	0.56	0.003	−0.23	0.22	0.010
BSM.MED	0.86	0.59	0.002	−0.23	0.21	0.012
FC.COM	0.67	0.59	0.002	−0.27	0.30	0.002
FB.COM	0.55	0.30	0.051	−0.15	0.09	0.115
FO.ON.MAX	0.76	0.56	0.003	−0.22	0.20	0.015
FO.WORLD.MAX	0.69	0.55	0.004	−0.24	0.23	0.008
FC.MAX	0.64	0.54	0.004	−0.24	0.22	0.010
FB.MAX	0.67	0.52	0.005	−0.24	0.23	0.008
FT.MAX	0.66	0.54	0.004	−0.26	0.25	0.006
BSM.MAX	0.98	0.61	0.002	−0.25	0.22	0.010
ROM.MAX	0.79	0.56	0.003	−0.23	0.20	0.016

Maximum species length recorded during field BsM indicated the weakest phylogenetic signal by all four indices (Table 3). This was the only metric not significantly different from random expectations by all indices. Average maximum length reported in the *Freshwater Fishes of Canada* and the average Ontario length reported in the *Freshwater Fishes of Ontario* were consistently among the metrics with the strongest phylogenetic signal across these indices. All metrics of length, other than the maximum length in BsM, indicated significant phylogenetic signal by all four indices.

**Table 3:** Four indices of phylogenetic signal and *P*‐values from randomization or likelihood ratio tests for significant phylogenetic signal in 12 measures of fish species length

	Moran's *I*		Abouheif's *C*_mean_		Pagel's *λ*		Blomberg's *K*	
	*I*	*P*	*C*	*P*	*λ*	*P*	*K*	*P*
FO.ON.AVG	0.28	0.012	0.35	0.011	1.09	<0.001	1.32	0.001
BSM.AVG	0.21	0.039	0.27	0.030	1.06	0.003	1.10	0.001
BSM.MED	0.24	0.028	0.32	0.015	1.07	<0.001	1.35	0.001
FC.COM	0.32	0.007	0.37	0.010	1.08	0.000	1.31	0.001
FB.COM	0.20	0.042	0.27	0.040	1.08	0.003	1.11	0.002
FO.ON.MAX	0.25	0.023	0.30	0.020	1.03	0.012	1.01	0.002
FO.WORLD.MAX	0.28	0.013	0.33	0.014	1.02	0.007	1.05	0.001
FC.MAX	0.25	0.024	0.29	0.022	1.01	0.017	0.99	0.001
FB.MAX	0.29	0.011	0.34	0.014	1.03	0.006	1.06	0.001
FT.MAX	0.30	0.010	0.35	0.013	1.03	0.005	1.09	0.001
BSM.MAX	0.13	0.117	0.18	0.090	0.97	0.104	0.81	0.003
ROM.MAX	0.27	0.014	0.33	0.017	0.99	0.011	1.06	0.001

## Discussion

Variability between sources in species trait data may influence the strength of traits as predictors and the ecological inferences we make using trait data. Intraspecific variation in trait values, both across a species range and within populations, could lead to variability in data collected at different scales and by different methods. Here, fish length varied with scale and type of metrics (e.g., average or maximum). Variation among metrics, however, was small in comparison with variation between species. Consistent with this finding, Blanck and Lamouroux (2006) reported smaller intra‐ than interspecific variation in length for European freshwater fishes. I also found consistent relative lengths across species reflected in high correlations between metrics (Fig. 2). Accordingly, changes in the type of metrics or the scale of the data source did not change the significance of length as an ecological predictor (with the exception of one metric, further discussed below). Average length in Ontario, however, explained the most variation in range boundary shifts across the province. Common length in Canada explained the most variation in the impacts of predator introductions to Ontario lakes. In my analysis, fish length is a reliable predictor of community‐level responses to environmental change. But, this may not be universal; trait reliability should be tested for other taxa or with additional species traits (Blanck and Lamouroux 2006; Kazakou et al. 2014).

I found relatively consistent phylogenetic signal in size data; however, this must be interpreted with care. First, while phylogenetic signal is significant, phylogenetic distance is clearly an imperfect predictor of fish size (Fig. 3C). Second, the species pool in the analyzed phylogeny, that of Manitoulin Island, is not a complete sample of the Great Lakes Basin or Ontario regional pool. Finally, while closely related species may share similar functional traits at a regional scale, indicated by phylogenetic signal, this may not hold true in a local community (Gerhold et al. 2015). Thus, the assumption of niche‐conservativism, which underlies the use of phylogenetic relatedness as a proxy for trait dispersion in community ecology, may best be tested using trait data sampled from local communities.

Several factors may influence the variability in species trait data between sources. Cordlandwehr et al. (2013) suggest the accuracy of species‐level traits retrieved from trait databases depends on three factors: the level of aggregation (scale), the plasticity of chosen traits, and habitat (environment). I add sampling bias as a fourth possible factor influencing the accuracy of trait data. Sampling bias may be location based or influenced by abundance, catchability or historical, cultural or economic interest in the species. For example, Sandel et al. (2015) found frequently measured plant species had higher trait values than rarely measured species. I found the maximum size of small prey fishes with intensive field sampling was often larger than the maximum recorded by other sources. In contrast, the maximum size of larger sportfishes in field sampling was often smaller than the maximum recorded by other sources. I suspect this is the result of historically poor sampling of small fishes with low catchability, the comparatively large samples of sportfish data by anglers and resource management agencies, and the reporting of large sportfish by anglers often during angling competitions. Ultimately, the variability in species trait data through all of these mechanisms is driven by intraspecific variation in traits.

Researchers should carefully evaluate species trait data for potential sampling or measurement biases and errors. Familiarity with the species included in community‐level analyses facilitates evaluating trait data. Additionally, outliers in the relationship between trait values taken from two different sources can indicate sampling bias or errors in transcription. For example, the median length of *Micropterus salmoides* (Largemouth Bass) in BsM field sampling (140 mm) is an outlier when compared to other metrics (Fig. 2, Supporting Information Appendix S1). In this case, the smaller than expected value for *M. salmoides* is due to a large number of juveniles sampled for this species. Average or median size metrics can be strongly influenced by juvenile life stages, whereas maximum observed metrics can reflect rarely observed sizes, those individuals on the tail end of the size distribution. In my analysis, the only metric by which length was not significantly related to range shifts or the impact of introduced predators was the common length reported in *FishBase*. The common length of *M. dolomieu* (Smallmouth Bass) reported by this database is 80 mm, which is clearly erroneous when compared with the values from other source (Fig. 2, Supporting Information Appendix S1). Tomelleri and Eberle (1990) are cited as the source of this value; however, they report a common length of 20 inches, suggesting a transcription error in the *FishBase* value. Replacing this value with 510 mm (~20 inches) would make the relationship between common length from *FishBase* and both range shifts and the impacts of introductions significant (*R* ^2^ = 0.42, *P* = 0.016 and *R* ^2^ = 0.25, *P* = 0.006, respectively).

As ecologists continue to incorporate species traits into analyses, it is important they consider potential sources of variability in data. Plant databases have begun to include quantitative environmental data for the location of sampled populations as well as population trait mean and variance (Violle et al. 2007; Kattge et al. 2011). This effort should be expanded across taxa and within aquatic ecosystems. Recently developed data‐collection tools and citizen‐science programs facilitate collecting trait data and location information with common standards (Duputié et al. 2014). In a period of rapid environmental change, species traits will continue to be important for predicting future changes in ecological communities. Accumulating measures of species traits from populations across environmental gradients will be needed to evaluate the relative importance of intraspecific variation and to understand how individual species will respond to environmental changes.

## Data Accessibility

Data are included in Supporting Information (See Below).

## Conflict of Interest

None declared.

## Supporting information


**Appendix S1.** Excel spreadsheet of twelve metrics of fish length collected from various sources for forty species (organized by length), range shift (degrees latitude) and predator risk (risk ratio) data, which species where included in the analyzed phylogeny and coefficient of variation (CV) across species and measures; see Table 1 for details of metrics and abbreviations.

## References

[ece32385-bib-0001] Albert, C. H. , W. Thuiller , N. G. Yoccoz , R. Douzet , S. Aubert , and S. Lavorel . 2010 A multi‐trait approach reveals the structure and the relative importance of intra‐ vs. interspecific variability in plant traits. Funct. Ecol. 24:1192–1201.

[ece32385-bib-0002] Albert, C. H. , F. de Bello , I. Boulangeat , G. Pellet , S. Lavorel , and W. Thuiller . 2012 On the importance of intraspecific variability for the quantification of functional diversity. Oikos 121:116–126.

[ece32385-bib-0003] Alofs, K. M. , and D. A. Jackson . 2015 The vulnerability of species to range expansions by predators can be predicted using historical species associations and body size. Proc. Biol. Sci. R. Soc. 282:20151211. 10.1098/rspb.2015.1211 PMC4528532 26180073

[ece32385-bib-0004] Alofs, K. M. , D. A. Jackson , and N. P. Lester . 2014 Ontario freshwater fishes demonstrate differing range‐boundary shifts in a warming climate. Divers. Distrib. 20:123–136.

[ece32385-bib-0005] Angert, A. L. , L. G. Crozier , L. J. Rissler , S. E. Gilman , J. J. Tewksbury , and A. J. Chunco . 2011 Do species' traits predict recent shifts at expanding range edges? Ecol. Lett. 14:677–689. 21535340 10.1111/j.1461-0248.2011.01620.x

[ece32385-bib-0006] de Bello, F. , S. Lavorel , S. Díaz , R. Harrington , J. H. C. Cornelissen , R. D. Bardgett , et al. 2010 Towards an assessment of multiple ecosystem processes and services via functional traits. Biodivers. Conserv. 19:2873–2893.

[ece32385-bib-0007] Blanck, A. , and N. Lamouroux . 2006 Large‐scale intraspecific variation in life‐history traits of European freshwater fish. J. Biogeogr. 34:862–875.

[ece32385-bib-0008] Bolnick, D. I. , P. Amarasekare , M. S. Araújo , R. Bürger , J. M. Levine , M. Novak , et al. 2011 Why intraspecific trait variation matters in community ecology. Trends Ecol. Evol., 26:183–192. 21367482 10.1016/j.tree.2011.01.009 PMC3088364

[ece32385-bib-0009] Brown, J. H. , J. F. Gillooly , A. P. Allen , V. M. Savage , and G. B. West . 2004 Toward a metabolic theory of ecology. Ecology 85:1771–1789.

[ece32385-bib-0010] Carlander, K. D. , and L. L. J. Smith . 1945 Some factors to consider in the choice between standard, fork, or total lengths in fishery investigation. Copeia 1945:7–12.

[ece32385-bib-0011] Cordlandwehr, V. , R. L. Meredith , W. A. Ozinga , R. M. Bekker , J. M. van Groenendael , and J. P. Bakker . 2013 Do plant traits retrieved from a database accurately predict on‐site measurements? J. Ecol. 101:662–670.

[ece32385-bib-0012] Doyle, B. C. 2013 Phylogenetic Structuring of Lake Fish Communities. University of Toronto. M.Sc, Thesis.

[ece32385-bib-0013] Duputié, A. , N. E. Zimmermann , and I. Chuine . 2014 Where are the wild things? Why we need better data on species distribution. Glob. Ecol. Biogeogr. 23:457–467.

[ece32385-bib-0014] Einum, S. , and I. A. Fleming . 2002 Does within‐population variation in fish egg size reflect maternal influences on optimal values? Am. Nat., 160:756–765. 18707463 10.1086/343876

[ece32385-bib-0015] Fenberg, P. B. , and K. Roy . 2008 Ecological and evolutionary consequences of size‐selective harvesting: how much do we know? Mol. Ecol. 17:209–220. 17868288 10.1111/j.1365-294X.2007.03522.x

[ece32385-bib-0016] Fitzsimmons, J. M. 2013 How consistent are trait data between sources? A quantitative assessment. Oikos 122:1350–1356.

[ece32385-bib-0017] Flynn, D. F. B. , N. Mirotchnick , M. Jain , M. I. Palmer , and S. Naeem . 2011 Functional and phylogenetic diversity as predictors of biodiversity–ecosystem‐function relationships. Ecology 92:1573–1581. 21905424 10.1890/10-1245.1

[ece32385-bib-0018] Gardner, J. L. , A. Peters , M. R. Kearney , L. Joseph , and R. Heinsohn . 2011 Declining body size: a third universal response to warming? Trends Ecol. Evol. 26:285–291. 21470708 10.1016/j.tree.2011.03.005

[ece32385-bib-0019] Gerhold, P. , J. F. Cahill , M. Winter , I. V. Bartish , and A. Prinzing . 2015 Phylogenetic patterns are not proxies of community assembly mechanisms (they are far better). Funct. Ecol. 29:600–614.

[ece32385-bib-0020] Gutowsky, L. F. G. , and M. G. Fox . 2012 Intra‐population variability of life‐history traits and growth during range expansion of the invasive round goby, *Neogobius melanostomus* . Fish. Manage. Ecol. 19:78–88.

[ece32385-bib-0021] Heins, D. C. , J. A. Baker , and J. M. Guill . 2004 Seasonal and interannual components of intrapopulation variation in clutch size and egg size of a darter. Ecol. Freshw. Fish 13:258–265.

[ece32385-bib-0022] HilleRisLambers, J. , P. B. Adler , W. S. Harpole , J. M. Levine , and M. M. Mayfield . 2012 Rethinking community assembly through the lens of coexistence theory. Annu. Rev. Ecol. Evol. Syst. 43:227–248.

[ece32385-bib-0023] Holm, E. , N. Mandrak , and M. Burridge . 2009 The ROM field guide to the freshwater fishes of Ontario. Royal Ontario Museum, Toronto, ON, Canada.

[ece32385-bib-0024] Jennings, S. , and J. L. Blanchard . 2004 Fish abundance with no fishing: predictions based on macroecological theory. J. Anim. Ecol. 73:632–642.

[ece32385-bib-0025] Kattge, J. , K. Ogle , G. Bönisch , S. Díaz , S. Lavorel , J. Madin , et al. 2011 A generic structure for plant trait databases. Methods Ecol. Evol. 2:202–213.

[ece32385-bib-0026] Kazakou, E. , C. Violle , C. Roumet , M.‐L. Navas , D. Vile , J. Kattge , et al. 2014 Are trait‐based species rankings consistent across data sets and spatial scales? J. Veg. Sci. 25:235–247.

[ece32385-bib-0027] Kelly, S. , R. Grenyer , and R. W. Scotland . 2014 Phylogenetic trees do not reliably predict feature diversity. Divers. Distrib. 20:600–612.

[ece32385-bib-0028] LaBarbera, M. 1989 Analyzing body size as a factor in ecology and evolution. Annu. Rev. Ecol. Syst. 20:97–117.

[ece32385-bib-0029] McGill, B. J. , B. J. Enquist , E. Weiher , and M. Westoby . 2006 Rebuilding community ecology from functional traits. Trends Ecol. Evol. 21:178–185. 16701083 10.1016/j.tree.2006.02.002

[ece32385-bib-0030] Meynard, C. N. , V. Devictor , D. Mouillot , W. Thuiller , F. Jiguet , and N. Mouquet . 2011 Beyond taxonomic diversity patterns: how do *α*,* β* and *γ* components of bird functional and phylogenetic diversity respond to environmental gradients across France? Glob. Ecol. Biogeogr. 20:893–903.

[ece32385-bib-0031] Mulder, C. , and J. J. Elser . 2009 Soil acidity, ecological stoichiometry and allometric scaling in grassland food webs. Glob. Change Biol. 15:2730–2738.

[ece32385-bib-0032] Münkemüller, T. , S. Lavergne , B. Bzeznik , S. Dray , T. Jombart , K. Schiffers , et al. 2012 How to measure and test phylogenetic signal. Methods Ecol. Evol. 3:743–756.

[ece32385-bib-0033] Ness, J. H. , J. L. Bronstein , A. N. Andersen , and J. N. Holland . 2004 Ant body size predicts dispersal distance of ant‐adapted seeds: implications of small‐ant invasions. Ecology 85:1244–1250.

[ece32385-bib-0034] Pakeman, R. J. 2014 Functional trait metrics are sensitive to the completeness of the species' trait data? Methods Ecol. Evol. 5:9–15.

[ece32385-bib-0035] R Development Core Team . 2014 R: a language and environment for statistical computing. R Foundation for Statistical Computing, Vienna, Austria Available at: http://www.R-project.org.

[ece32385-bib-0036] Sandel, B. , A. G. Gutiérrez , P. B. Reich , F. Schrodt , J. Dickie , and J. Kattge . 2015 Estimating the missing species bias in plant trait measurements. J. Veg. Sci. 26:828–838.

[ece32385-bib-0037] Sandstrom, S. , M. Rawson , and N. Lester . 2010 Manual of instructions for broad‐scale fish community monitoring; using large mesh gillnets and small mesh gillnets. Ontario Ministry of Natural Resources, Peterborough, ON.

[ece32385-bib-0333] Scott, W. B. , and E. J. Crossman . 1998 Freshwater fishes of Canada. Galt House Publications Ltd, Oakville, ON Canada.

[ece32385-bib-0038] Swenson, N. G. , P. Anglada‐Cordero , and J. A. Barone . 2011 Deterministic tropical tree community turnover : evidence from patterns of functional beta diversity along an elevational gradient. Proc. R. Soc. B 278:877–884. 10.1098/rspb.2010.1369 PMC3049044 20861048

[ece32385-bib-0039] Tomelleri, J. R. , and M. E. Eberle . 1990 Fishes of the central United States. University Press of Kansas, Lawrence, KS.

[ece32385-bib-0040] Violle, C. , M.‐L. Navas , D. Vile , E. Kazakou , C. Fortunel , I. Hummel , et al. 2007 Let the concept of trait be functional!. Oikos 116:882–892.

[ece32385-bib-0041] Violle, C. , B. J. Enquist , B. J. McGill , L. Jiang , C. H. Albert , C. Hulshof , et al. 2012 The return of the variance: intraspecific variability in community ecology. Trends Ecol. Evol. 27:244–252. 22244797 10.1016/j.tree.2011.11.014

[ece32385-bib-0042] Winemiller, K. O. , and K. A. Rose . 1992 Patterns of life‐history diversification in North American fishes: implications for population regulation. Can. J. Fish Aquat. Sci. 49:2196–2218.

[ece32385-bib-0043] Woodward, G. , B. Ebenman , M. Emmerson , J. M. Montoya , J. M. Olesen , A. Valido , et al. 2005 Body size in ecological networks. Trends Ecol. Evol. 20:402–409. 16701403 10.1016/j.tree.2005.04.005

